# The role of contextual materials in object recognition

**DOI:** 10.1038/s41598-021-01406-z

**Published:** 2021-11-09

**Authors:** Tim Lauer, Filipp Schmidt, Melissa L.-H. Võ

**Affiliations:** 1 Scene Grammar Lab, Department of Psychology, Goethe University Frankfurt, Theodor-W.-Adorno-Platz 6, PEG 5.G144, 60323 Frankfurt am Main, Germany; 2 Department of Experimental Psychology, Justus Liebig University Giessen, 35394 Giessen, Germany; 3 Center for Mind, Brain and Behavior (CMBB), University of Marburg and Justus Liebig University, Giessen, Germany

**Keywords:** Human behaviour, Object vision, Perception

## Abstract

While scene context is known to facilitate object recognition, little is known about which contextual “ingredients” are at the heart of this phenomenon. Here, we address the question of whether the materials that frequently occur in scenes (e.g., tiles in a bathroom) associated with specific objects (e.g., a perfume) are relevant for the processing of that object. To this end, we presented photographs of consistent and inconsistent objects (e.g., perfume vs. pinecone) superimposed on scenes (e.g., a bathroom) and close-ups of materials (e.g., tiles). In Experiment 1, consistent objects on scenes were named more accurately than inconsistent ones, while there was only a marginal consistency effect for objects on materials. Also, we did not find any consistency effect for scrambled materials that served as color control condition. In Experiment 2, we recorded event-related potentials and found N300/N400 responses—markers of semantic violations—for objects on inconsistent relative to consistent scenes. Critically, objects on materials triggered N300/N400 responses of similar magnitudes. Our findings show that contextual materials indeed affect object processing—even in the absence of spatial scene structure and object content—suggesting that material is one of the contextual “ingredients” driving scene context effects.

## Introduction

We almost never see objects in isolation, but virtually always within a rich visual context. This context is not random but particular objects tend to occur in particular spaces or “scene contexts” with varying likelihoods. For instance, a pot is more likely encountered in the kitchen than in the bathroom and a fire hydrant will rather be seen on the street than in the living room. Additionally, objects tend to occur at particular locations within these spaces, for example, the pot is rather sitting on the stove than on the kitchen floor. Over the course of our lifetime, we have acquired knowledge of these co-occurrences of objects, scenes and locations within scenes that help us, among other things, in searching for and recognizing objects^1–3^.

For example, early behavioral studies used line drawings in a forced choice paradigm to show that a consistent object within its scene context (e.g., a fire hydrant on the street) is detected faster and more accurately compared to an inconsistent object (e.g., a sofa on the street)^4–6^. However, these early studies were criticized for not taking response biases into account^7,8^ (for a review, see^9^). Therefore, in recent years, scene context effects were investigated using an object naming paradigm, where observers type in the name of an object after seeing it for a short time superimposed on (or embedded in) a naturalistic scene background^10–15^. Across all of these studies, consistent objects on scenes were named more accurately than inconsistent ones (“scene consistency effect”)—which cannot be explained in terms of response bias or similarity in low-level features or shape between objects and scenes^11^. Rather, the effect appears to be driven by the meaning of objects and scenes. In a recent study from our group^13^, we found that the scene consistency effect has two components: Consistent objects superimposed on scenes yielded higher object naming accuracies compared to consistent objects on meaningless scrambled scenes (baseline), suggesting that scene context *facilitates* object recognition. Conversely, inconsistent objects on scenes yielded lower naming accuracies compared to inconsistent objects on scrambled scenes (baseline), indicating an *interference* with object naming performance.

In addition to behavioral measures, context effects on object processing have been studied using electroencephalography (EEG), with different components of event-related potentials (ERPs) associated with different processes. For example, the *N400* is a well-known component that is sensitive to semantic violations in language^16,17^ but also to violations in other domains including scene perception. Specifically, inconsistent objects in scenes trigger a more negative potential than consistent objects with a mid-central maximum approximately 400 ms after stimulus onset^13,14,18–22^. The N400 is thought to reflect object-scene semantic processing on a conceptual level (see^21^; see also^18,19^; for a review see^23^). Most studies have also reported scene context effects in an earlier time window of the ERPs^13,14,18–20,22^, and it has been argued that this *N300* component reflects context effects on a more perceptual level. Specifically, the component might represent the cost of matching incoming visual information of an inconsistent object with context-based predictions, before the completion of object identification^18,19^. In line with this explanation, a recent ERP study directly manipulated consistency and object identifiability to provide evidence that the N300 indexes context effects on object identification^20^. Note, however, that the distinction between the N300 and the N400 component based on their underlying processes is still a matter of debate^24^.

While it is well known that scene context can boost object recognition, and we start to understand the temporal characteristics and neural correlates of this process, little is known about which contextual “ingredients” are at the heart of scene context effects. Specifically, naturalistic scenes contain a wealth of information potentially relevant for object processing. For example, scenes may contain other objects—indeed, it has been shown that objects next to other, related objects are named more accurately than objects next to unrelated objects^25^ (see also^26^). At the same time, scene context effects on object recognition will also occur without deriving meaning from other objects in a scene. By definition, scenes are spaces^27^, and certain categories of spaces have certain perceptual properties. The spatial envelope model can distinguish scenes computationally based on their spatial structure (i.e., their spatial layout) without taking object identities into account^28^. Behavioral studies have demonstrated that these global scene properties are processed very rapidly, and allow us to grasp a scene’s meaning or “gist” even after very brief image exposure (e.g.,^29^). Critically, there is evidence that the spatial structure of scenes affects object recognition. Objects primed with a semantically consistent global ensemble texture—which is preserving spatial layout information of the original scene but no object semantics—are named more accurately than objects primed with an inconsistent texture^30^. Also, distorting the global configuration of scene photographs by randomly re-arranging the scene’s parts in a grid (4 × 4 or 8 × 8) results in lower object recognition performance (^15^; see also^31^). Finally, scene inversion (i.e., rotation of the image by 180 degrees) was shown to affect context effects on object recognition^13^.

These studies provide first insights into which contextual “ingredients” might be useful for object recognition, particularly co-occurring objects and the spatial scene structure. However, there is another important source of information that has not yet been explored and might be useful for object recognition: contextual materials^32,33^. Many scenes are “made of” certain types of materials. For instance, asphalt is common in a street scene but not in a bathroom. Conversely, ceramics are frequently encountered in a bathroom but not in a street. Naturally, objects are surrounded by these materials (e.g., a tube of toothpaste by the ceramics of the sink). Indeed, in close-up views of objects, co-occurring object and scene structure information is often reduced or not accessible so that contextual materials might be particularly relevant and aid object recognition. While prior research has not addressed the question of whether *contextual* materials influence object recognition, there is extensive work on the role of materials in object recognition. Specifically, studies demonstrated the significant contributions of color and surface structure, together with object shape, on the recognition of objects (e.g.,^34–39^).

**Figure 1:**
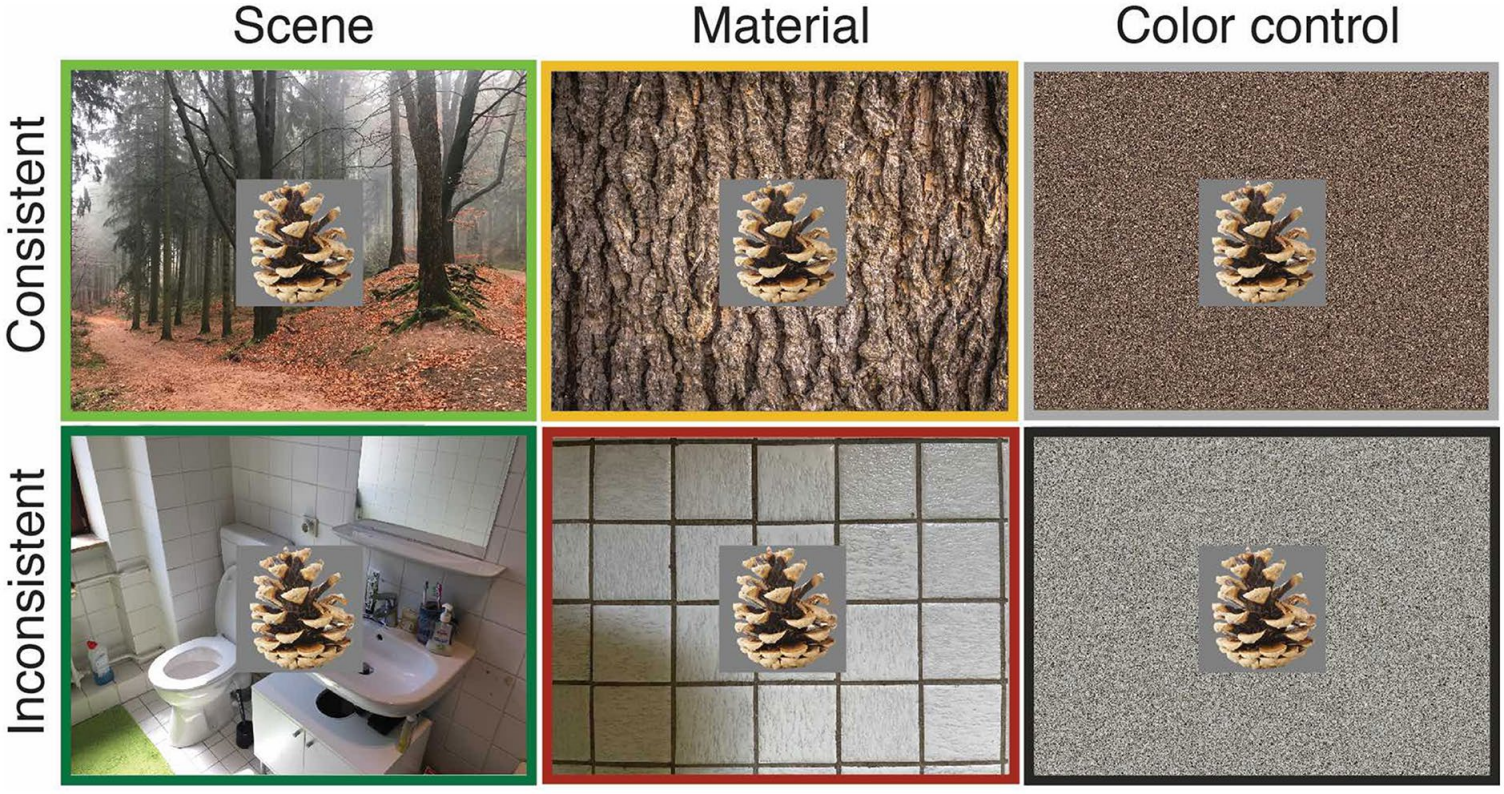
Example stimuli from the six experimental conditions of Experiment 1. Note that in Experiment 2, the color control condition (scrambled materials) was not included to maximize the number of trials in the other conditions. Colored frames were added for illustration purposes.

Here, we examined whether contextual materials that frequently occur in particular scenes influence object processing in a similar way as these scenes have been shown to. In Experiment 1, we briefly presented consistent and inconsistent thumbnail objects (i.e., isolated objects on gray backgrounds) superimposed on three types of background images: scenes, materials, and scrambled materials (color control condition) (Fig. 1). Materials were chosen based on a survey in which an independent group of participants freely reported the dominant materials for a number of scene categories. We hypothesized that if contextual materials modulate object recognition as scenes do, consistent objects superimposed on materials should be named more accurately than inconsistent ones. To examine whether a potential effect would be driven by the color information in the materials, we included scrambled material images that preserved the global color information but eliminated diagnostic surface structure. We predicted that mere color information would not differentially affect object naming performance. In addition, we addressed the question of whether materials would facilitate and/or interfere with object recognition. Facilitation should be reflected in increased naming accuracy for consistent objects on materials compared to consistent objects on scrambled materials (baseline). Interference, on the other hand, should be reflected in decreased accuracy for inconsistent objects on materials compared to inconsistent objects on scrambled materials (baseline). In Experiment 2, we recorded event-related potentials as a complementary, temporally precise measure of context effects on object processing. Consistent and inconsistent objects were presented superimposed on scenes and materials. To ensure that the stimuli were attended, participants completed a Repetition Detection cover-up Task (RDT)^22^. Again, if materials modulate semantic object processing just like scenes, there should be N300/N400 responses for inconsistent versus consistent objects on materials—just like observed in scenes (e.g.,^13^).

## Results

### Experiment 1

**Figure 2:**
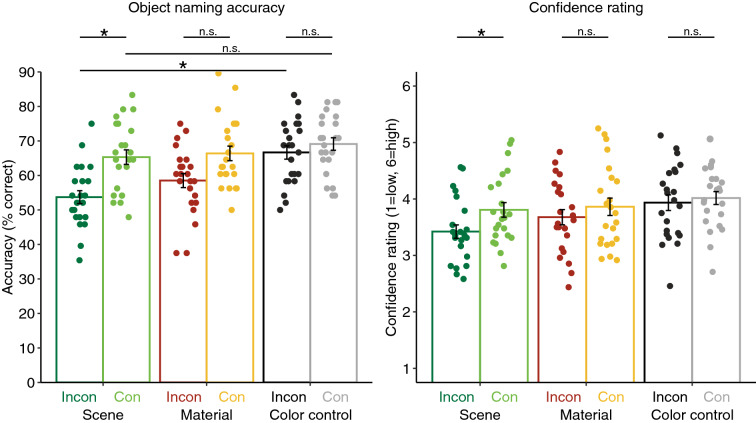
Object naming accuracy (left panel) and confidence rating (right panel) for inconsistent (Incon) and consistent (Con) objects superimposed on scenes, materials, and color controls (scrambled materials), respectively. Data points represent individual participants’ means; bars represent the means per condition. Error bars depict the standard error of the means. Asterisks indicate significant comparisons (*p* < 0.05); non-significant comparisons are marked with “n.s.”.

Figure 2 shows object naming accuracy (left panel) and confidence (right panel) for all conditions. For the statistical analysis, we submitted the single-trial accuracy (correct/incorrect) and confidence rating (from 1 = low to 6 = high) separately to generalized linear mixed-effects models (GLMMs) (for more details, see “Data analysis” section and^13^). We conducted three planned comparisons per model: consistent and inconsistent objects were contrasted per background type (scene, material, scrambled material). In addition, we tested for main effects of scenes and materials compared to scrambled materials (baseline) and for a main effect of consistency. Then, we tested for interactions between scenes and scrambled materials, using treatment contrasts for consistency (consistent vs. inconsistent), and between materials and scrambled materials. Finally, we performed post-hoc tests for all significant interactions to evaluate whether there was facilitation and/or interference of object naming performance (by scenes and/or materials) compared to the baseline (scrambled materials).

#### Object naming accuracy

Planned contrasts for consistent versus inconsistent objects on scenes yielded a significant difference in object naming accuracy, *ß* = 0.596, *SE* = 0.204, *z*_ratio_ = 2.925, *p* = 0.003. For consistent versus inconsistent objects on materials, we found a marginal but non-significant difference, *ß* = 0.388, *SE* = 0.205, *z*_ratio_ = 1.894, *p* = 0.058, and no difference for scrambled materials, |z_ratio_|< 1. Compared to scrambled materials (baseline), we did not find main effects for scenes, *ß* = − 0.201, *SE* = 0.111, *z* = − 1.805, *p* = 0.071, or materials, *ß* = − 0.164, *SE* = 0.154, *z* = − 1.065, *p* = 0.287. The main effect of consistency was not significant either, |z|< 1. However, we found an interaction between scrambled materials and scenes with respect to the consistency manipulation, *ß* = − 0.47, *SE* = 0.139, *z* = − 3.373, *p* < 0.001, and a marginal but non-significant interaction between scrambled materials and materials with respect to the consistency manipulation, *ß* = − 0.262, *SE* = 0.141, *z* = − 1.859, *p* = 0.063. Following up on the significant interaction found for scenes (with scrambled materials), we tested if there was a facilitation and/or interference of object naming performance for scenes compared to scrambled materials (baseline): Post-hoc tests showed no significant difference between consistent objects on scenes versus consistent objects on scrambled materials, *ß* = 0.201, *SE* = 0.111, *z*_ratio_ = 1.805, *p* = 0.071, but a significant difference between inconsistent objects on scenes versus inconsistent objects on scrambled materials, *ß* = 0.670, *SE* = 0.109, *z*_ratio_ = 6.129, *p* < 0.001.

#### Confidence ratings

Planned contrasts for consistent versus inconsistent objects superimposed on scenes showed a significant difference in confidence ratings, *ß* = 0.11, *SE* = 0.043, *z*_ratio_ = 2.536, *p* = 0.011, but we did not find a significant difference for consistent versus inconsistent objects on materials, *ß* = 0.054, *SE* = 0.043, *z*_ratio_ = 1.239, *p* = 0.215, or scrambled materials, |z_ratio_|< 1. Compared to scrambled materials (baseline), we did not find main effects for scenes, *ß* = − 0.056, *SE* = 0.03, *z* = − 1.85, *p* = 0.064, or materials, *ß* = − 0.045, *SE* = 0.036, *z* = − 1.234, *p* = 0.217. The main effect of consistency was not significant either, |z|< 1. However, there was an interaction between scrambled materials and scenes with respect to the consistency manipulation, *ß* = − 0.089, *SE* = 0.031, *z* = − 2.869, *p* = 0.004, but no interaction between scrambled materials and materials with respect to the consistency manipulation, *ß* = − 0.032, *SE* = 0.031, *z* = − 1.055, *p* = 0.292.

### Experiment 2

**Figure 3:**
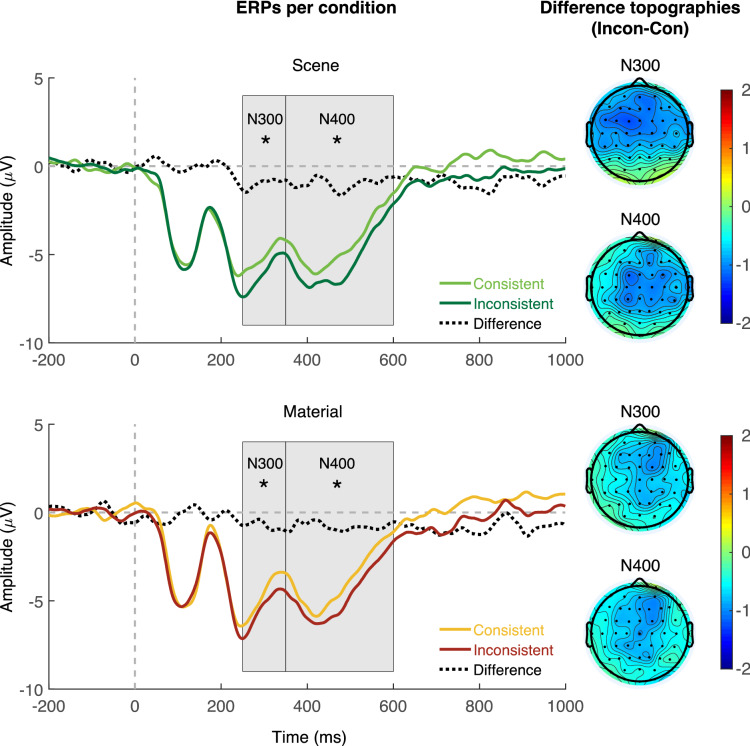
Grand-averaged ERPs for the mid-central region (electrodes FC1, FCz, FC2, C1, Cz, C2, CP1, CPz, CP2) for consistent versus inconsistent objects on scenes (top panel) and materials (bottom panel), as well as corresponding difference topographies for inconsistent versus consistent objects in the N300 and N400 time windows.

Figure 3 shows the grand-averaged ERPs per condition (consistent scene, inconsistent scene, consistent material, inconsistent material) for the mid-central region as well as topographies of the difference between N300/N400 ERPs for consistent and inconsistent objects per background type (scene, material). Descriptively, inconsistent objects on scenes evoked a more negative N300/N400 ERP compared to consistent objects, with topographies indicating a distribution of the negativity over fronto-central electrodes. Critically, inconsistent versus consistent objects on materials evoked a similar albeit weaker negativity in the N300/N400 time window.

For the statistical analysis, single-trial ERP amplitudes in the N300 and N400 time window per condition were extracted and separately submitted to linear mixed-effects models (LMMs). For both time windows of interest, we conducted planned comparisons between consistent and inconsistent objects per background type (scene, material). Moreover, we examined whether there are main effects of consistency (consistent, inconsistent) and background type (scene, material), as well as a possible interaction of the two factors.

#### RDT performance

Performance on the cover-up task (RDT) was generally high: On average, participants scored 13.18 out of 16 possible hits (i.e., exact repetitions of intermixed object-scene pairings were correctly identified; *min* = 8, *max* = 16). The average number of false alarms was 2.05 out of 32 possible false alarms (for more information on RDT trials, see “Procedure” section).

#### N300 time window

In the N300 time window, planned contrasts for consistent versus inconsistent objects showed a significant difference for scenes, *ß* = 0.980, *SE* = 0.347, *t*_*ratio*_ = 2.824, *p* = 0.005, and for materials, *ß* = 0.784, *SE* = 0.345, *t*_*ratio*_ = 2.271, *p* = 0.023. Moreover, we found a main effect of consistency, *ß* = − 0.98, *SE* = 0.347, *t* = − 2.824, *p* = 0.005, but no main effect of background type, *ß* = 0.439, *SE* = 0.376, *t* = 1.165, *p* = 0.249, and no interaction of the two factors, |*t*|< 1.

#### N400 time window

In the N400 time window, planned contrasts for consistent versus inconsistent objects yielded a significant difference for scenes, *ß* = 0.962, *SE* = 0.338, *t*_*ratio*_ = 2.848, *p* = 0.004, and materials, *ß* = 0.815, *SE* = 0.336, *t*_*ratio*_ = 2.423, *p* = 0.015. Moreover, we found a main effect of consistency, *ß* = − 0.962, *SE* = 0.338, *t* = − 2.848, *p* = 0.004, but no main effect of background type, *ß* = 0.588, *SE* = 0.471, *t* = 1.249, *p* = 0.233, and no interaction of the two factors, |*t*|< 1.

## Discussion

While numerous studies have shown that scene context influences object recognition, little is known about which contextual “ingredients” scene context effects are based on. The aim of this study was to examine whether object recognition is affected by contextual materials^32,40^ occurring in scenes.

In Experiment 1, we briefly presented consistent and inconsistent objects superimposed on scenes, materials, and scrambled materials (which served as color controls for the materials). We found that consistent objects on *scenes* were named more accurately and with higher confidence than inconsistent ones, replicating the well-known scene consistency effect^10–14^. For consistent versus inconsistent objects on *materials*, we found a marginal non-significant difference in object naming accuracy in the same direction and no effect in confidence ratings. For the color control condition (scrambled materials), we did not observe any effect of the consistency manipulation on either accuracy or confidence ratings. Finally, we examined if the consistency effect found for scenes can be decomposed into facilitation and/or interference: When contrasting accuracies for consistent objects on scenes and scrambled materials (baseline), we did not find a significant difference and thus no indication that object recognition was *facilitated*. By contrast, when comparing accuracies for inconsistent objects on scenes and scrambled materials (baseline), we found a difference, indicating that there was *interference*.

In Experiment 2, we presented consistent and inconsistent objects superimposed on scenes and materials. We recorded ERPs as a complementary temporally precise measure of context effects. For scenes, we found N300/N400 responses, that is, inconsistent objects on scenes elicited a more negative potential than consistent ones. This is in line with previous studies reporting N300/N400 effects for objects on (or in) scenes^13,14,18–20,22,24^. Critically we also found N300/N400 responses of similar magnitudes for materials, suggesting that they exerted a contextual influence just like scenes.

This N400 effect for inconsistent versus consistent objects superimposed on materials may signal semantic processing on a conceptual level, just as proposed for semantic violations in scenes^18,19^ or in other domains like language^23^. Specifically, the effect might arise from difficulties to integrate inconsistent objects with their material context (relative to consistent objects). While the N400 response is sensitive to various types of semantic violations in multiple domains, the N300 has been associated with the processing of complex objects and scenes specifically^41^. There is evidence that the N300 is linked to object identification: Both a manipulation of object-to-scene consistency and object identifiability was shown to elicit an N300 effect but ERPs for inconsistent objects differed from ERPs for unidentifiable objects later than consistent objects did^20^. In line with this, in a recent study, the N300 was proposed to index predictive coding and perceptual hypothesis testing^41^: Seeing representative (typical) exemplars of scenes resulted in decreased N300 amplitudes compared to less representative scene images—even though there were no semantic violations in the scene photographs. In our study, the N300 for inconsistent versus consistent objects on materials may thus reflect contextual modulation on a perceptual level of object processing—before object identification is completed—at a stage, where incoming visual information of a stimulus is matched with prior knowledge^18,19^. Specifically, inconsistent material backgrounds may have produced (misleading) predictions of object identity, which were matched with bottom-up input of the stimulus at a higher cost compared to consistent conditions. In addition to these recent accounts, Hamm and colleagues^42^ found indication that the N300 reflects early pre-identification categorization and not necessarily semantic access like the N400: When priming objects with words that either represented a basic or a subordinate category (e.g., “bird” vs. “raven”), an N300 effect was only observed with subordinate primes followed by an object from a different basic category (e.g., car) but not when followed by a mismatching object from a different subordinate category (e.g., pigeon). In contrast, an N400 effect was observed irrespective of whether the mismatch occurred at a basic or subordinate level. Even though the N300 thus appears to be less sensitive to “identify” object incongruency compared to the N400, we found an N300 effect for materials—which might imply categorical effects of materials prior to object identification^42^. However, it should be noted that the precise mechanisms underlying the N300 and N400 responses are still debated, as well as whether the two components can be clearly distinguished^24^.

Together, the ERP effects suggest that materials exerted contextual influence with respect to the critical objects. Note that the material backgrounds in our study were close-up photographs of materials lacking spatial layout information or recognizable objects. This implies that contextual influences on object processing can arise even in the absence of spatial scene structure (e.g. depth or spatial layout information) and co-occurring objects that have previously been shown to modulate object recognition^25,26,30^. In this regard, our findings are in line with a recent study from our group in which we also found that global scene properties may affect object processing even in the absence of spatial layout information and object semantics^14^: Inconsistent versus consistent objects superimposed on scene textures—that preserved global scene summary statistics but no spatial layout information or recognizable objects—evoked similar albeit weaker N300/N400 responses as original scenes. Note that the whole scene served as input for synthesizing artificial textures in this study, whereas, in the current study, we used close-up photographs of selected real materials.

### Limitations and future directions

It is somewhat surprising that we did not find a significant consistency effect for materials in the behavioral paradigm (Experiment 1) based on the accuracies for consistent and inconsistent materials (Fig. 2). We note that our best-fitting statistical model accounted for variability between material categories through a random slope for category (for more details, see “Data analysis” section). In an exploratory fashion, we simplified the model by removing the random slope and found that the comparison between accuracies for consistent and inconsistent materials was statistically highly significant, *ß* = 0.396, *SE* = 0.0968, *z*_ratio_ = 4.086, *p* < 0.001, whereas there was still no consistency effect for scrambled materials (*p* = 0.231). A regular paired t-test also yielded a highly significant difference for consistent versus inconsistent materials, *t*(22) = − 4.012, *p* < 0.001. Given these exploratory results, we suggest that different material categories might produce behavioral context effects of different magnitudes. However, the design of the current study is arguably not suitable for investigating context effects for individual material categories (e.g., sand) or subsets of categories (e.g., outdoor materials). Critically, besides a low number of trials, the counterbalancing of stimuli was not intact for subsets of categories. Nonetheless, for exploratory investigations, we calculated context effects separately for indoor and outdoor backgrounds (see supplementary S1, S2, S3). Indeed, we found that, in the behavioral paradigm, the effect is strongly driven by outdoor backgrounds and not existent for indoor backgrounds (see S1). In the EEG paradigm (S2 and S3), the effect appears to be stronger for indoor compared to outdoor materials. Again, note that these differences between indoor and outdoor backgrounds have to be interpreted with caution given that consistency was manipulated by pairing each indoor and outdoor object with an indoor *and* outdoor background (in order to control for systematic differences between consistent and inconsistent objects, e.g., in low-level properties). This control for systematic stimulus differences is lost when evaluating context effects separately for indoor and outdoor backgrounds. Overall, our study was not designed to look at individual material categories and their effects on object perception. However, our exploratory observations suggest that future studies should examine context effects as a function of scene and material category, for example, by using a balanced design in which each object is shown both in a consistent and inconsistent setting in the categories of interest.

Finally, it might be somewhat surprising that we did not find indication of contextual facilitation in Experiment 1 when contrasting accuracies for consistent objects on scenes and scrambled materials (baseline). Some previous studies have reported facilitation effects when scene context was present versus absent (e.g.,^13,15,43^), yet others have not found such effects (e.g.,^10,14,44^). Possibly, these mixed results are due to stimulus characteristics (for a review, see^9^). While the thumbnail objects used in the current study yielded a high degree of control, figure-ground segmentation was arguably easier for these objects than for embedded objects, which may have weakened or diminished contextual facilitation of object recognition. Moreover, the baseline (scrambled materials) may have contained some useful information (e.g., color of sand), possibly reducing the magnitude of contextual facilitation for scenes.

In future endeavors, new developments in machine learning^45^ might allow us to figure out the different contributions to context effects by linking the effects in human observers to the activations in different layers of deep neural networks. By comparing activations for individual objects and individual scenes or materials, we will be able to generate predictions about which combinations are similar (dissimilar) and would therefore be expected to produce higher (lower) accuracies in human observers. By testing whether the observed effects are better explained by similarities of activations in lower versus higher levels of the neural networks, we will be able to produce testable hypotheses about the nature of the respective effects (e.g., rather low-level stimulus features or high-level semantic features).

## Conclusions

Taken together, our findings show that materials—the “stuff” that scenes and objects are “made of”—are one of the ingredients of scenes that feed into context effects on object processing, in addition to known effects of spatial layout information and co-occurring objects. Learning these and other statistical regularities of our environment over a lifetime allows us to flexibly draw on different sources of information, depending on availability, to efficiently recognize objects in scenes.

## Method

### Participants

Twenty-three participants completed Experiment 1 (18 females, *M* = 20.91 years old, *SD* = 2.68) and 22 different participants completed Experiment 2 (13 females, *M* = 23.27 years old, *SD* = 3.59). All had normal or corrected-to-normal vision (at least 20/25 acuity), were unfamiliar with the stimuli, and received course credit or payment. In Experiment 1, four additional participants were excluded because of a programming error resulting in multiple missing trials (*N* = 2), because instructions were not followed (*N* = 1), or because German was not the native language (*N* = 1) which was a requirement in Experiment 1. In Experiment 2, two additional participants were excluded: One participant scored zero hits in the RDT and one had poor vision and did not follow instructions. The number of participants was based on previous studies using the same experimental paradigm and methods^13,14^. Written informed consent was obtained at the beginning of the experiment. All aspects of the data collection and analysis were carried out in accordance with guidelines approved by the Human Research Ethics Committee of the Goethe University Frankfurt.

### Stimuli and design

**Figure 4:**
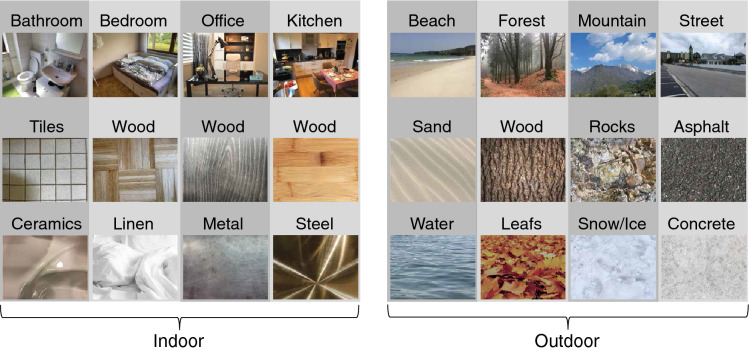
Scene and material categories that were used in the experiments. Top row: example stimuli from the four indoor and outdoor scene categories. Middle and bottom row: example stimuli of the two dominant materials per scene category.

We collected 144 photographs of indoor scenes and 144 photographs of outdoor scenes (1024 × 768 pixels) from several categories in equal numbers (kitchen, office, bathroom, bedroom, mountain, beach, forest, street) using Google image search and the LabelMe database^46^. In addition, we collected 144 photographs of indoor materials and 144 photographs of outdoor materials, assigned to two classes of materials per scene category as outlined below. The materials were chosen based on a survey conducted at Goethe University Frankfurt, where 22 students independently stated which two materials would most likely occur in the given scene categories. Participants were given an example of a material and a corresponding scene category that was not included in the stimulus set. Then, we determined which two materials were reported most frequently per category after discarding synonyms (e.g., ceramics and porcelain were considered the same type of material): (1) bathroom: tiles, ceramics; (2) bedroom: wood, linen; (3) office: wood, metal; (4) kitchen: wood, steel; (5) beach: sand, water; (6) forest: wood, leaves; (7) mountain: rocks, snow; and (8) street: concrete, asphalt. Figure 4 provides an illustration of the scene and material categories that were used. We collected several exemplars of these two types of materials per category (e.g., tiles of various sizes and colors), while striving to choose exemplars that fitted the respective scenes well—for example, we chose natural wood for the forest category and processed wood for the kitchen category. All photographs depicted close-up views of materials, thereby not including any “scene-like” images with spatial layout information^28^. Further, we did not include images that contained any recognizable objects or more than one type of material, or uniform images without any surface structure.

Each scene was paired with a semantically consistent thumbnail object (e.g.,^47–50^) on a gray background (256 × 256 pixels). In order to manipulate semantic consistency, we paired indoor and outdoor scenes such that each object was consistent with one scene and inconsistent with another (see Fig. 1). Note that the majority of object-scene pairs was identical to the ones used in previous work^13,14^ but some pairs were replaced in order to further improve the quality of the stimulus set. Then, each object was paired with an indoor and outdoor material that would be consistent or inconsistent with the object. In order to control for any influence of the color of the materials, each object was paired with a scrambled version of the paired material which was generated by randomly re-arranging the pixels in the material image. All background images (scenes, materials, scrambled materials) were randomly assigned to the six experimental conditions (consistent scene, inconsistent scene, consistent material, inconsistent material, consistent scrambled material, inconsistent scrambled material) and counter-balanced across participants using a 3 × 2 Latin square design (see also^13,14^). Note that in Experiment 2, we excluded the scrambled materials condition, resulting in a 2 × 2 Latin square design. One advantage of the Latin square design is that background images and objects are not presented more than once throughout the experiment per participant. For Experiment 1, we generated a dynamic perceptual mask using the Masked Priming Toolbox^51^.

### Apparatus and EEG recording

The Stimuli were shown on a 24-inch monitor with a refresh rate of 144 Hz at a viewing distance of approximately 60 cm. This resulted in visual angles of 26.56° horizontally and 20.03° vertically for background images (scenes, materials, scrambled materials), and 6.75° both horizontally and vertically for thumbnail objects. The experiment was programmed and conducted using MATLAB and the Psychophysics Toolbox^52,53^. The EEG was recorded in a shielded cabin at a sampling rate of 1000 Hz using 62 active electrodes which were positioned on the scalp according to the 10–20 system (amplifier: actiChamp, Brain Products, Germany). Two additional electrodes placed on the mastoids served as references. Moreover, an electrode positioned below the left eye served as an EOG channel.

### Procedure

#### Experiment 1

**Figure 5:**
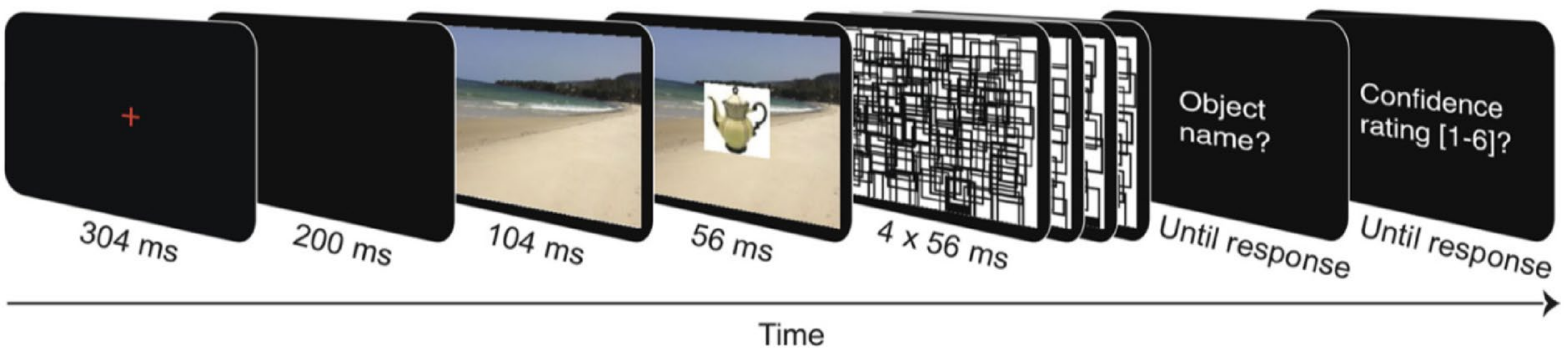
Trial sequence of Experiment 1 (object naming experiment).

The procedure is similar to the one used in a previous study^13^. Participants completed six practice trials and 288 main trials. In the trial sequence (Fig. 5), a central fixation cross was presented for 304 ms, followed by a blank screen for 200 ms, a preview of the background image (scene, material, or scrambled material) for 104 ms, the critical object (consistent or inconsistent) superimposed on the same background image for 56 ms, a dynamic perceptual mask (4 × 56 ms), and finally an input panel where participants entered the name of the critical object. Participants were instructed to name the object as precisely as possible using a single German word (e.g., “apple” instead of “fruit”) (see also^13,14^). Participants were instructed to provide their best guess in case they missed the object or were uncertain about the correct name. The object naming response was finalized when pressing the return key. Then, a rating panel was shown, prompting participants to judge how confident they were about their response on a scale from one (low) to 6 (high). To initiate the next trial, participants pressed any key. Participants were instructed to look at the center of the screen throughout the trial (but not while being presented with the input panels).

#### Experiment 2

**Figure 6:**
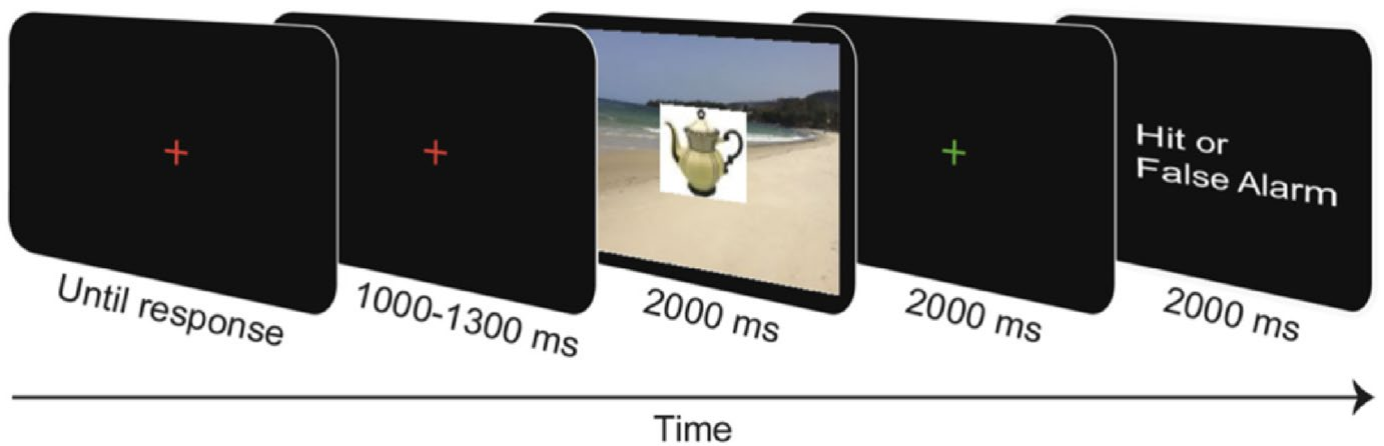
Trial sequence of Experiment 2 (ERP experiment). Note that the last frame, “Hit or False Alarm” was only presented if a key was pressed in the response window of the RDT (indicated by the green fixation cross). If no key was pressed in the response window, the participants received feedback on misses but not on correct rejections.

Participants completed six practice trials and 336 main trials which consisted of 288 experimental trials and 48 intermixed Repetition Detection Task trials (RDT) (for a similar procedure, see^13,14^). The purpose of the RDT trials was to ensure that participants attended the stimuli^22^. The trial sequence was as follows (see Fig. 6). In the beginning of each trial, a red central fixation cross was presented, encouraging participants to blink if necessary. Once participants pressed any key, the fixation cross was shown for another 1000–1300 ms, followed by the presentation of the critical object (consistent or inconsistent) superimposed on a background image (scene or material) for 2000 ms. Participants were instructed not to blink during this period and to look at the center of the screen. Subsequently, a green fixation cross was shown for 2000 ms, indicating the response window of the RDT: Participants were instructed to press a key if they spotted an exact repetition, that is, if they had already seen the same object-background combination in any previous trial but not when spotting a novel object-background combination or a lure (i.e., when only the object but not the background was repeated or vice versa). If a key was pressed during the response window, there was visual feedback on whether the response was a hit or a false alarm. If no key was pressed, there was only feedback on misses, not on correct rejections. Repetitions occurred up to ten trials after first presentation of an object-background combination. All stimuli that were shown as part of the RDT (16 exact object-background repetitions, 8 lures) were not part of the main stimulus set. RDT trials were excluded from the ERP analysis.

### Preprocessing

#### Experiment 1

Two independent raters (undergraduate students at Goethe University Frankfurt) that were blind to condition scored each response as correct or incorrect based on a sample solution. Raters were instructed to score responses correct if they matched the sample solution or one of its synonyms (e.g., car or automobile). Correct spelling was not a criterion for scoring. Importantly, responses that were not as descriptive as the sample solution (e.g. “fruit” instead of “apple”) were supposed to be considered incorrect by the raters (see^10^; see also^13,14^). All responses for which raters disagreed were settled by a third independent rater (undergraduate student), making the final decision.

#### Experiment 2

The continuous EEG data was bandpass filtered (0.1–45 Hz) and notch filtered (50 Hz) offline. We conducted an Independent Components Analysis (ICA) on a copy of the data that was filtered 1–45 Hz and segmented into 4500 ms epochs, time-locked to stimulus onset (ranging from − 900 to + 3600 ms). Before calculating the ICA, we removed noisy electrodes (identified through visual inspection) and large non-stereotypical artefacts (defined as epochs in which the signal exceeded an absolute voltage threshold of + /− 500 microvolt at any channel) from these datasets. The resulting ICA weights were transferred to the original 0.1–45 Hz filtered continuous data. We then removed EOG components from the data using ICLabel^54^, an EEGLAB plugin for automatic component classification based on machine learning. Specifically, we removed all components that were classified as EOG with a probability of > 0.5 and that unlikely reflected brain activity (probability < 0.05) (*M* = 2.3 components; *min* = 0, *max* = 4). We interpolated those electrodes that had been removed before conducting the ICA (noisy electrodes). Then, we segmented the continuous data into 1200 ms epochs, time locked to stimulus onset (− 200 to 1000 ms) and applied baseline correction by subtracting the average signal preceding stimulus onset. All trials that were part of the RDT (*N* = 48 out of 336 epochs per participant) were discarded. We also removed experimental trials in which a false alarm occurred (*M* = 1.77, *min* = 0, *max* = 17 epochs out of 288 experimental epochs per participant). Further, we removed all epochs that contained EEG artefacts using a reproducible semi-automatic procedure. Specifically, we tailored an absolute voltage threshold and a moving window peak-to-peak threshold to each participant’s data (for a similar approach, see^13,14^). Epochs in which the amplitude exceeded at least one of these thresholds (at one or more electrodes) were removed. On average, 66.82 out of 72 epochs per condition were retained (*min* = 46).

Subsequently, we calculated the mean N300 (250–350 ms) and N400 (350–600 ms) amplitude in the mid-central region (averaging across electrodes FC1, FCz, FC2, C1, Cz, C2, CP1, CPz, CP2). The time windows and region of interest were chosen based on previous research demonstrating robust scene context effects on object processing (see^22^; see also^13,14,24^). Grand-averaged ERPs per condition (Fig. 3) were low-pass filtered at 30 Hz for display purposes. The data was preprocessed in EEGLAB^55^ and ERPLAB^56^.

### Data analysis

The statistical analysis is similar to the analysis in^13^. Single-trial data were subjected to mixed-effects models using lme4^57^, a library for the programming environment R^58^. As fixed effects, each model included the type of background image (Experiment 1: scene, material, scrambled material; Experiment 2: scene, material) and consistency (consistent, inconsistent). The random effects structure was maximal at first^59^, with random intercepts for participants, scene categories, and items (individual images) as well as random slopes for participants and scene categories. Since models with random intercepts and slopes for all fixed effects often fail to converge or result in overparameterization, we simplified the random effects structure using the following procedure^13,14^: We used Principal Components Analysis (PCA) of the random-effects variance–covariance estimates for each fitted mixed-effects model in order to find cases of overparameterization; random slopes that were not supported by the PCA and did not contribute significantly to the goodness of fit in likelihood ratio tests were removed. Note that we first removed item-related slopes (i.e., scene category), and then participant-related slopes if necessary^13^. The final best-fitting model structure is reported below. Note that all statistics were calculated on these best-fitting models and that all models were fit using maximum likelihood estimation.

#### Experiment 1

For both dependent variables (object naming accuracy, confidence ratings) the best-fitting model included random intercepts for participants, scene categories, and items as well as by-category random slopes for background type (scene, material, scrambled material) and consistency (consistent, inconsistent). Both models were GLMMs with a Binomial distribution (object naming accuracy) or Poisson distribution (confidence ratings). Post-hoc tests for significant interactions were *p*-value adjusted using the Holm-correction (R package lsmeans^60^).

#### Experiment 2

The best-fitting model for the N300 time window included random intercepts for participants, scene categories, and items as well as a by-participant random slope for background type (scene, material). The best-fitting model structure for the N400 time window was identical except that it additionally included a by-category random slope for background type (scene, material). *P*-values for both LMMs were calculated using Satterthwaite’s degrees of freedom method (R packages lmerTest, lsmeans, and emmeans^60,61^).

## Supplementary Information

Supplementary Information.

## Data Availability

The data and analysis scripts are available on the Open Science Framework: https://osf.io/nqz4t/?view_only=4ac9d6c53d864624bee3d28c07009628.
