# Supplementary material for: The role of contextual materials in object recognition
Source: Sci Rep. 2021 Nov 9;11:21988. doi: 10.1038/s41598-021-01406-z (PMC8578445; doi:10.1038/s41598-021-01406-z)
Supplement: Supplementary file 1 — Supplementary Information. [file 41598_2021_1406_MOESM1_ESM.pdf]

Supplementary Information

Supplementary S1: Accuracy for indoor and/or outdoor backgrounds

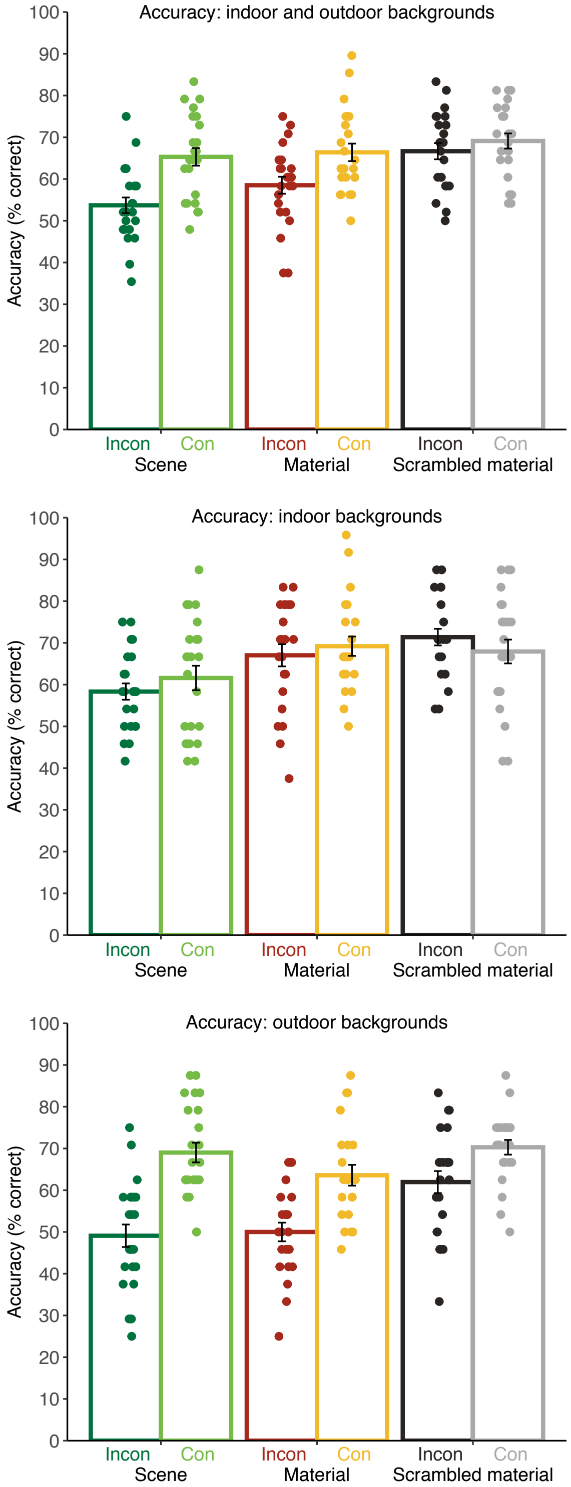

Figure S1. Object naming accuracy for inconsistent (Incon) and consistent (Con) objects on indoor and/or outdoor backgrounds (Scenes, Materials, Scrambled materials). Note that only when considering both indoor and outdoor backgrounds (top panel), the counterbalancing of stimuli was intact (i.e., each indoor and outdoor object was paired with an indoor *and* outdoor background). By contrast, in the case of indoor backgrounds (middle panel), all consistent objects were indoor objects while all inconsistent objects were outdoor objects. In the case of outdoor backgrounds (bottom panel) all consistent objects were outdoor objects while all inconsistent objects were indoor objects.

Supplementary S2: N300 amplitude for indoor and/or outdoor backgrounds

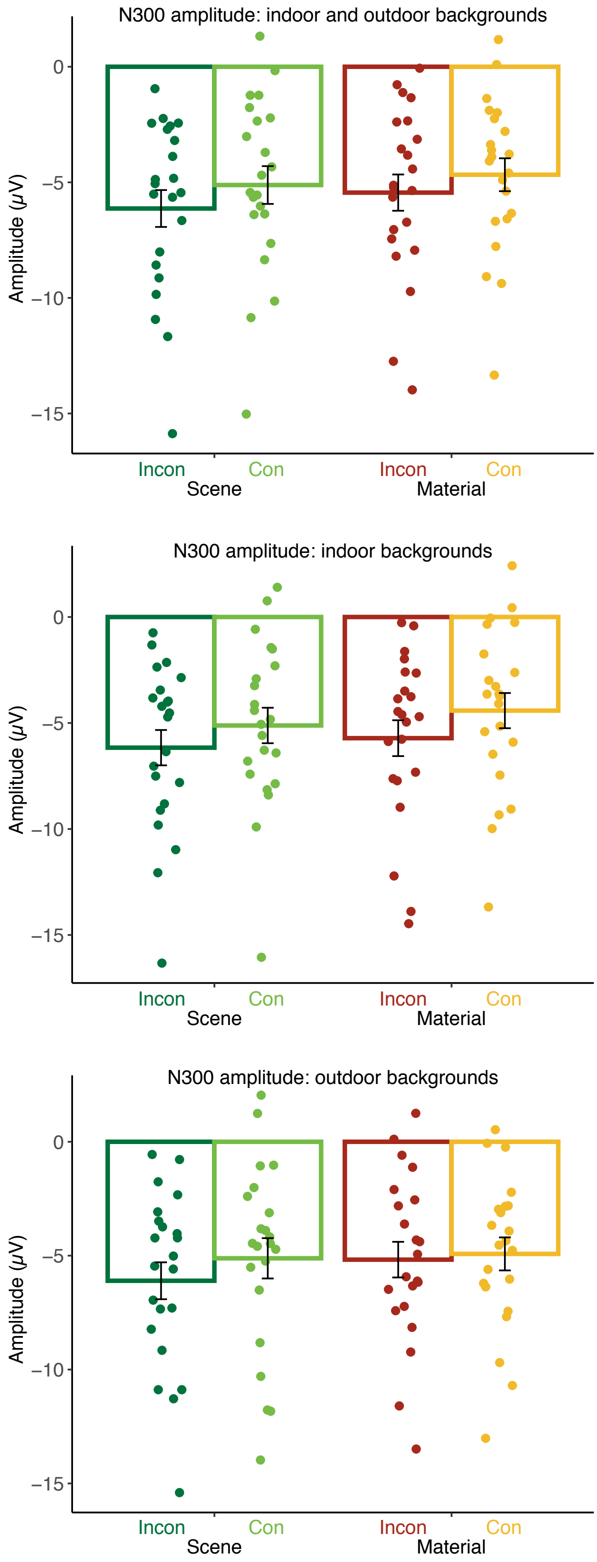

Figure S2. N300 amplitude for inconsistent (Incon) and consistent (Con) objects on indoor and/or outdoor backgrounds (Scenes, Materials). Note that only when considering both indoor and outdoor backgrounds (top panel), the counterbalancing of stimuli was intact (i.e., each indoor and outdoor object was paired with an indoor *and* outdoor background). By contrast, in the case of indoor backgrounds (middle panel), all consistent objects were indoor objects while all inconsistent objects were outdoor objects. In the case of outdoor backgrounds (bottom panel) all consistent objects were outdoor objects while all inconsistent objects were indoor objects.

Supplementary S3: N400 amplitude for indoor and/or outdoor backgrounds

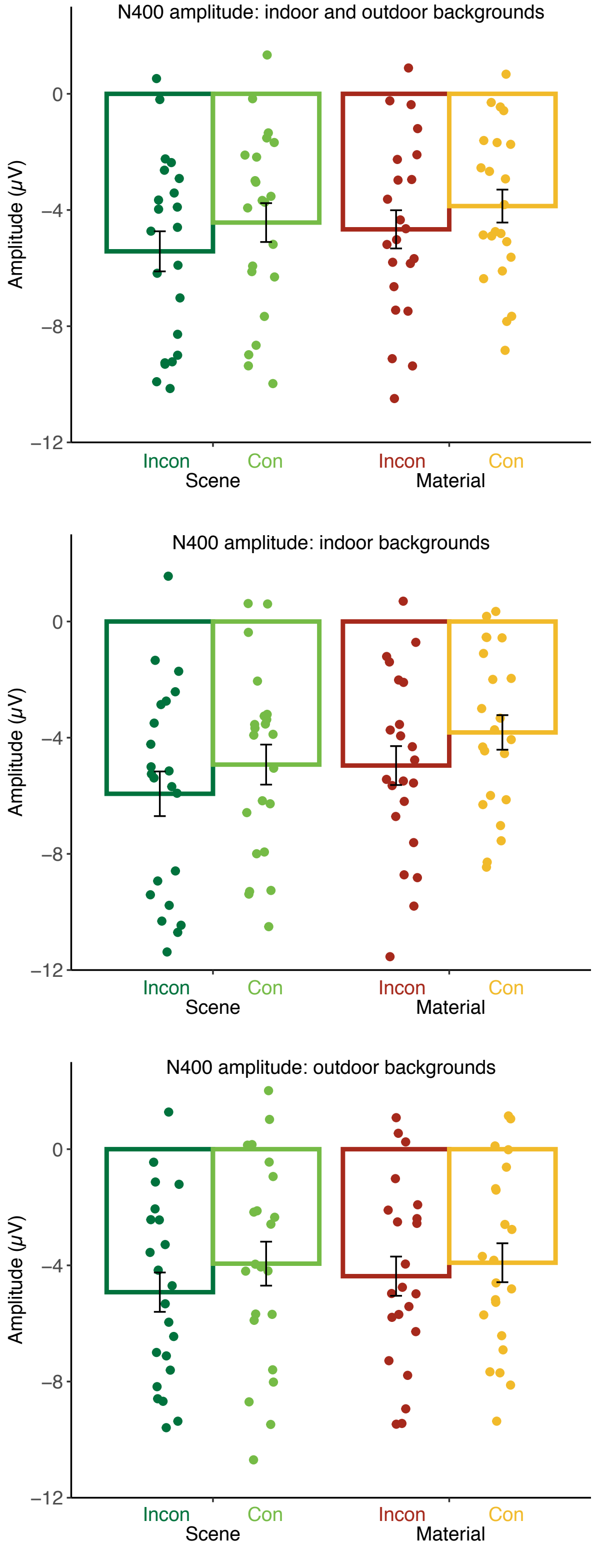

Figure S3. N400 amplitude for inconsistent (Incon) and consistent (Con) objects on indoor and/or outdoor backgrounds (Scenes, Materials). Note that only when considering both indoor and outdoor backgrounds (top panel), the counterbalancing of stimuli was intact (i.e., each indoor and outdoor object was paired with an indoor *and* outdoor background). By contrast, in the case of indoor backgrounds (middle panel), all consistent objects were indoor objects while all inconsistent objects were outdoor objects. In the case of outdoor backgrounds (bottom panel) all consistent objects were outdoor objects while all inconsistent objects were indoor objects.
